# Dynamics of *Vibrio ostreicida* in *Mytilus galloprovincialis* through *in vivo* infections

**DOI:** 10.3389/fimmu.2025.1727164

**Published:** 2025-11-26

**Authors:** Martina Leonessi, Jose R. Lopez, Yanis Cruz-Quintana, Manon Auguste, Karl B. Andree, Margarita Fernandez-Tejedor, Luigi Vezzulli, Dolors Furones, Laura Canesi

**Affiliations:** 1 Department of Earth, Environmental and Life Sciences (DISTAV), University of Genoa, Genoa, Italy; 2 National Biodiversity Future Center, Palermo, Italy; 3 IRTA, Aquaculture Program, La Ràpita, Spain; 4 Universidade de Santiago de Compostela, Santiago de Compostela, Spain; 5 Facultad de Acuicultura y Ciencias del Mar, Universidad Técnica de Manabí, Bahía de Caráquez, Ecuador

**Keywords:** *V. ostreicida* isolate, mussel mortality, cohabitation infection, bath infection, PCR, horizontal transmission, hemolymph responses, histopathology

## Abstract

**Introduction:**

*Vibrio species* are widespread in coastal environments, where they are being increasingly associated with mortality episodes of farmed bivalves. This is the case for *Vibrio ostreicida* strain r172 isolated from a mortality event of adult mussels *Mytilus galloprovincialis* in Spain in 2022.

**Methods and Results:**

In this study, the infection dynamics and immune responses of adult *M. galloprovincialis* challenged with *V. ostreicida* r172 were investigated using different *in vivo* experimental approaches. First, a virulence assay by injection (1 x 108 CFU/100 µL) was performed at different temperatures (12, 18 and 24 °C). The results showed that mussel mortality (about 50 % within 8 days) was independent of increasing temperatures. Subsequently, an injection/cohabitation experiment was carried out placing together in the same tank *V. ostreicida*-injected (Donors, 108 CFU/mL) with un-injected mussels (Recipients). The time course of infection was then followed by evaluating positivity to *V. ostreicida* by PCR, responses of haemolymph components (haemocyte lysosomal membrane stability and serum lysozyme activity) and tissue histopathology (gills and digestive gland). The results showed a partial horizontal transfer of *V. ostreicida* from infected to uninfected mussels, with transient effects on haemolymph responses and histopathological lesions in both groups. Finally, in order to mimic more realistic environmental conditions, a bath infection experiment was carried out, exposing mussels to *V. ostreicida* in seawater (105 CFU/mL). This condition resulted in lower stress in haemocytes; moreover, no lysozyme release or histopathological alterations were observed.

**Discussion:**

Overall, the results show that *M. galloprovincialis* is able to cope with challenge with *V. ostreicida*, indicating that this Vibrio species is moderately pathogenic to adult mussels under the established experimental conditions.

## 1 Introduction

The genus *Vibrio* represents one of the most diverse and important group of bacterial pathogens found in marine and coastal waters at the global scale. Some *Vibrio* species are responsible for a wide range of human infections *via* direct exposure to seawater or through the consumption of seafood, including farmed bivalves (1). In the last decade, *Vibrio* infections have been described as one of the main cause of diseases affecting all life stages of different species of bivalves (2–4). Recurrent mortality episodes may lead to the complete loss of production stocks, with serious economic consequences (5).

Different *Vibrio* species have been described as etiological agents of vibriosis that promote larval and spat mortalities of different bivalve species in hatcheries worldwide (6). Among them, *V. aestuarianus* has emerged as an important pathogenic species responsible of mass mortalities of spat and juveniles of the Pacific oyster (*Magallana gigas*) in France (7–10). Pathogenic infections of larvae of mussels, clams, scallops and oyster spat due to *V.* sp*lendidus*-related strains have been demonstrated as well (11–15). *V. coralliilyticus* was associated with disease outbreaks in multiple host larvae species including the oysters *M. gigas* and *Ostrea edulis*, the hard clam *Mercenaria*, and the Atlantic Bay scallop *Argopecten irradians* (6, 16, 17).

Adult bivalves can also be affected by mortality outbreaks. Mass mortality events of clams (*Venerupis philippinarum*) leading to serious economic losses in France and Spain have been associated with Brown Ring Disease (BRD) caused by *V. tapetis* (9). Among vibrios, *V. aestuarianus* has been identified as the main putative cause of oyster mortality in France since 2012 (3, 10), Spain (18), Ireland (19) and the United Kingdom (20).

Bivalves belonging to *Mytilus* spp., in particular *M. edulis* and *M. galloprovincialis*, have been so far considered to be very resistant to bacterial infections, in particular vibriosis, due to their particularly efficient innate immune system (4, 21). However, in the last decade, the frequency of mortality events of adult mussels, often associated with microbial infection, has dramatically increased in both natural and farmed populations (22). Indeed, during these events, different *Vibrio* strains, all from the *Splendidus* clade, have been isolated from moribund animals (3, 23–26).

However, little is known of infection kinetics of potentially pathogenic Vibrio species in mussels. Studying the interactions between vibrios and the immune system of the bivalve hosts may help preventing and predicting bacterial infections (27–30). In this context, understanding infection dynamics is an essential step in developing disease management strategies (14, 31).

*V. ostreicida* is an oyster larval pathogen first isolated from a nursery of flat oysters (*Ostrea edulis*) suffering from repeated disease outbreaks in Galicia (32, 33). A *V. ostreicida* strain r172 was recently isolated from a mortality episode of adult *M. galloprovincialis* that occurred in April 2022 in Alfacs Bay (Tarragona, Spain) (34). The phylogenetic analysis placed *V. ostreicida* r172 within a clade that included *Vibrio* species such as *V. coralliilyticus* and *V. neptunius*, both known for representing potential threats to mussel adults and larvae (12, 16). However, further studies are needed to evaluate the *in vivo* pathogenicity of this strain in adult mussels.

In the present work, the infection dynamics and immune responses of adult *M. galloprovincialis* challenged with the *V. ostreicida* strain r172 were investigated using different experimental protocols. We first carried out a virulence assay *via* injection at different water temperatures to evaluate the role of this key environmental variable in the outcome of infection. Subsequently, a model cohabitation experiment between infected and uninfected mussels was performed in order to investigate the possibility of horizontal transfer of bacteria among individuals. The time course of infection was followed in injected (Donors) and non-injected (Recipients) mussels by evaluating presence of *V. ostreicida* by PCR, responses of hemolymph components (in terms of hemocyte lysosomal stability and lysozyme activity), and histopathological conditions. Finally, a bath infection was performed to simulate a more natural environmental infection scenario, and the same parameters were evaluated.

## 2 Materials and methods

### 2.1 Bacterial cultures and inoculum preparation

The *V. ostreicida* strain *r172* utilized in this work was isolated from an adult mussel mortality episode that occurred in April 2022 in an aquaculture farm in Alfacs Bay, Ebro River Delta, (Tarragona, Spain) (34) close to the research center of IRTA- La Ràpita. *V. ostreicida r172* was grown on marine agar (MA) plates at 23°C for 24h and some colonies resuspended in PBS (phosphate buffered solution, pH 7.4) to obtain an Abs_550_ of about 0.8. Bacteria were then harvested by centrifugation (4,500 x *g* for 10min) and resuspended in 5 mL of PBS to obtain a final concentration of 10^9^ CFU/mL. The final concentration was also determined by total viable counts on MA plates after 24h growth at 23°C. Marine agar (MA) was used for all culture preparations throughout the experiments.

### 2.2 Animals

Adult mussels (4–5 cm long) from local farms (IRTA), were collected in February 2023 (for virulence assay), and in May 2024 and July 2024 (for cohabitation and immersion experiments, respectively). Animals were transferred to the laboratory and acclimatized in static tanks containing aerated ASW (1 L/animal), pH 7.9–8.1, 35 ‰ for 24h at 18°C, unless otherwise indicated.

### 2.3 *In vivo* virulence assay at different temperatures (February 2023)

Experiments were carried out on adult mussels (5cm on average) purchased in February 2023 from Fangar Bay (Explotacions Marines, La Ràpita) at a seawater temperature of 12.8°C. Four groups of 10 mussels each (40 animals per condition) were acclimatized to three different temperatures: 12–14.5°C; 18–19.5°C; 23.5–24.5°C. After one week, two groups of mussels kept at each temperature were challenged with *V. ostreicida* by intravalvar injection (1 x 10^8^ CFU/100 µL; 100 µL/mussel), and the other two were injected with PBS (control group). Therefore, data were obtained on two groups of 10 mussels each for each condition (20 mussels each). During the 14 days of the trial, each group was kept at the same acclimatization temperature. Mortality was checked daily. Dead mussels were removed and whole tissue homogenates of each individual were plated on MA at 23 °C for 24h. Detection of *V. ostreicida* among the bacterial colonies grown on the plates was carried out using the PCR protocol previously described (34), using 400 ng of DNA in each PCR reaction.

### 2.4 Detection of *V. ostreicida* in mussel samples from different aquaculture farms by real time PCR (April 2024)

A screening of mussel stocks from both Ebro bays (Alfacs and Fangar Bays) and from nearby open waters (Cases de Alcanar, Tarragona, Spain) (**Supplementary Figure S1**) was carried out in April 2024 to choose those with the lowest *V. ostreicida* background load for subsequent laboratory infection experiments. Adult mussels sampled from different sites were transferred to the laboratory, and 30 individuals were analyzed for detection of *V. ostreicida* by PCR, following the protocol described in (34), with slight modifications (see details in **Supplementary Table S1** and **Supplementary Table S2**). PCR analysis was applied to either bacterial cultures from hemolymph pools or individual mantle as described below.

Hemolymph was extracted from the posterior adductor muscle using a sterile 1 mL syringe with an 18 G1/2” needle, filtered through sterile gauze and pooled in a Falcon tube at 18°C (35); 6 pools of derived from 5 animals each were made (n = 6). Aliquots of 100 µL of each hemolymph pool were plated on MA and incubated for 48h at 23°C to allow for bacterial growth. The same procedure was applied to single aliquots (100 µL) of seawater sampled from each tank at each time point. At the end of incubation, samples were collected into Eppendorf tubes. Using a 1 µL loop, a small quantity of samples from each tube was gathered and resuspended in 100 µL of Milli-Q water. DNA extraction was performed by incubating the resuspended samples for 5min at 95–99°C in a thermocycler; samples were then immediately put in ice, centrifuged for 5min at 8,000 x g at 4°C and the supernatants collected for PCR analyses. In both hemolymph and seawater bacterial cultures, PCR analyses allow for the determination of live, growing bacteria.

PCR analysis was also directly applied to mantle tissue to reveal the total bacterial load (live and dead bacteria). Individual mantle samples (n = 30) were dissected, frozen at -80°C, and were directly analyzed for the presence of *V. ostreicida*. DNA was extracted from 30 mg tissue using the DNeasy Blood & Tissue kit (Qiagen) according to the manufacturer’s instructions. The DNA concentration was measured using a NanoDrop 2000.

The same sample preparation and PCR protocols were subsequently used for evaluating the presence of *V. ostreicida* in mussel samples from *in vivo* challenge experiments (before challenge, at day 0, and at different time points during each experiment). Data are reported as % of positive PCR samples with respect to the total number of samples analyzed.

### 2.5 Mussel infection by injection and water tank cohabitation model (May 2024)

Mussels obtained from Alcanar Bay in May 2024 (at a seawater T = 18.7°C) were injected into the posterior adductor muscle with 50 µL of bacterial suspension of *V. ostreicida* (10^8^ CFU/mL final nominal concentration), as previously described in studies on determination of mussel immune responses to other vibrios (27, 36), as well as in previous cohabitation studies in oysters and mussels (31, 37–39). Injected mussels, hereafter indicated as Donors (D), were placed in the same tank filled with UV-treated and filtered seawater (0.5 L/animal), with un-injected animals, hereafter indicated as Recipients (R).A preliminary cohabitation experiment was performed testing different ratios between D and R in a total of 80 mussels for each tank (1D:1R, 1D:3R and 1D:10R, see **Supplementary Figure S2**). Different groups were separated in small netted containers in each tank. The experiment was run twice.

**Figure 1:**
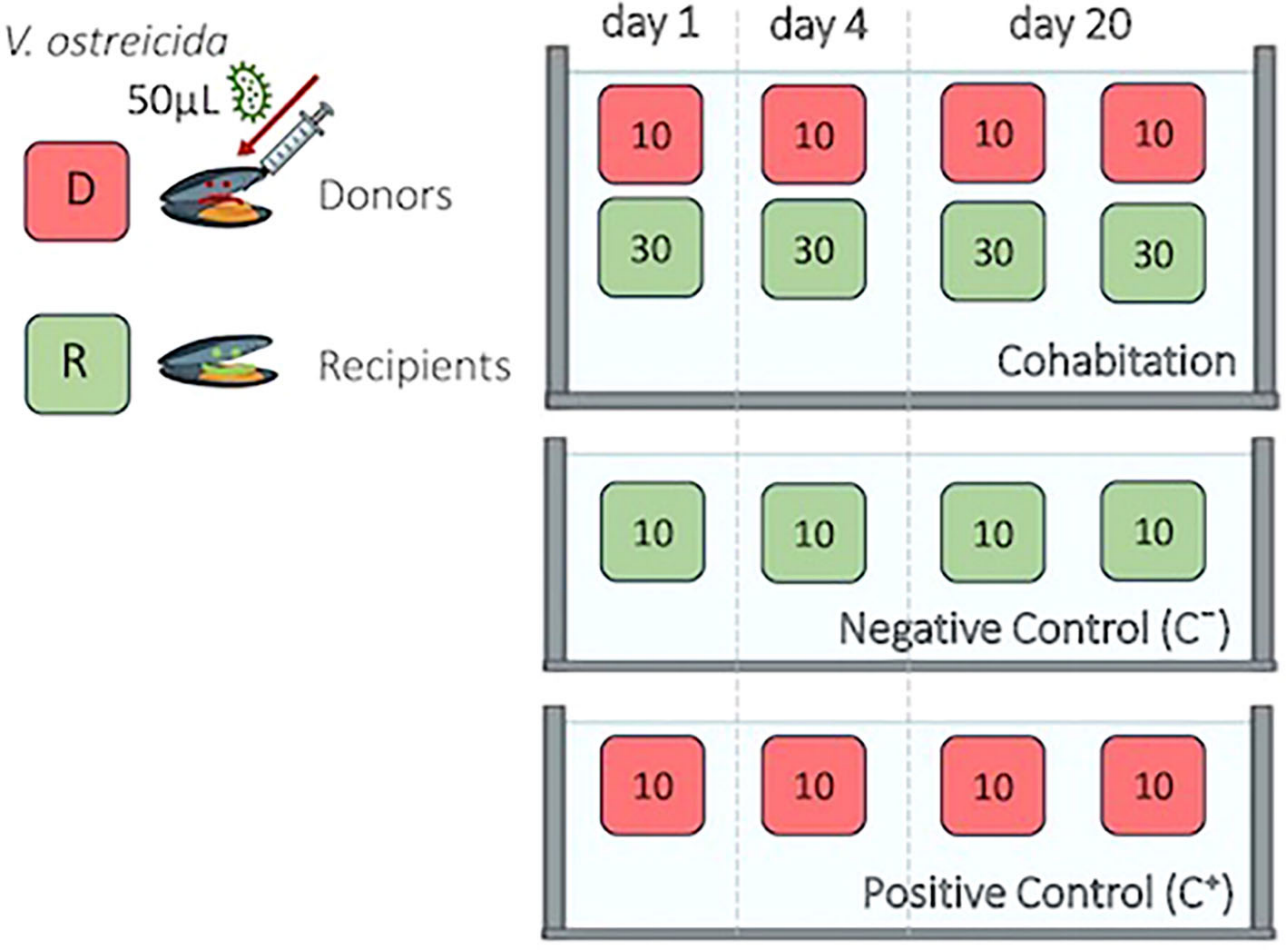
Cohabitation experimental set up.

Since no significant mortality was observed in any experimental condition (see results **Supplementary Figure S3**), the intermediate ratio 1D:3R was chosen for final experiments (see **Figure 1**) as previously described (31, 39). In the cohabitation tank, a total of 160 mussels were thus utilized (40 D:120 R). Donors D were divided into 4 subgroups of 10 animals each. Recipients R were split into 4 subgroups of 30 animals each. Positive (*V. ostreicida*-injected) (C^+^) controls and negative (PBS injected) controls (C^-^) were run in parallel (4 subgroups of 10 animals each, for a total of 40 mussels).

Mussels were sampled from each tank at 1, 4 and 20 days post injection (p.i.). During the experiment, animals were maintained under static conditions at 18°C with aeration and without feeding. Mortality was monitored daily, and aliquots of 100 mL of SW were sampled from each tank before the daily water change.

At each time point, individual mantles were collected and frozen at -80°C (n = 10 for C^-^, C^+^ and D; n = 30 for R). Aliquots of water from tanks (100 µL) and of hemolymph pools (100 µL) were plated on MA for subsequent PCR analysis. Replicate samples were made of 3 pools of hemolymph from 3 mussels each (n = 3) for C^-^ and C^+^ and D groups, and 6 hemolymph pools from 5 animals each (n = 6) for R, respectively. At each time point, individual histological sections were collected (n=10 for C^-^, C^+^ and D samples; n = 30 for R samples).

### 2.6 Mussel infection by immersion (July 2024)

In July 2024, another experiment was performed of mussel infection with *V. ostreicida* by water immersion, using mussels sampled from Alcanar (330 individuals). Seawater T at the day of sampling was 24.9°C). Before the experiment, the presence of *V. ostreicida* was evaluated in 30 animals by PCR as described above).

After 24h acclimatization at 18°C, a suspension of *V. ostreicida* (final nominal concentration 10^5^ CFU/mL) was spiked once into the water tank (150 animals) at day 0. A parallel group of control (unexposed) mussels was kept in clean seawater (150 animals). Mussels were divided into subgroups inside smaller netted containers within each tank (5 subgroups of 30 animals each). Seawater was changed daily with no addition of bacteria. Before daily water change, animals were fed with phytoplankton *Tetraselmis suecica* (200.000 cell/mL in the tank) in order to stimulate the filtration process. Mortality was checked daily in a group of 30 mussels for each condition. Mussels from all tanks (1 subgroup condition) were sampled at 1, 2, 3 and 4 days post infection. Hemolymph, mantle and water were collected. Individual mantles were dissected and frozen at -80 °C for PCR analysis (n = 30 for each experimental group). Hemolymph pools of 5 animals each were made (n = 6 for each experimental group). Individual mantles were dissected and frozen at –80°C for PCR analysis (n = 30 for each experimental group). Tissue samples for histopathological analysis were also collected and fixed as described below. All sampling procedures and assays were performed as described above for the cohabitation experiment.

### 2.7 Mussel hemolymph parameters

In both injection and immersion experiments, in all experimental conditions the remaining hemolymph from each pool was utilized for determination of functional parameters (16, 35, 40). Lysosomal membrane stability (LMS) was evaluated as an indicator of cellular stress in immune cells, the hemocytes, using the Neutral Red Retention Time assay (NRRT); lysozyme activity was determined spectrophotometrically as a marker of immune related enzymes in serum (see Methods in SI for experimental details). LMS and lysozyme activity were evaluated in 3 hemolymph pools for C^-^, C^+^ and D (n = 3) and 6 pools for R samples (n = 6) for the cohabitation experiment and in 6 pools for each condition (n = 6) for the bath infection.

### 2.8 Histopathological analysis

For histopathological evaluation, a transverse section (0.3 cm) including digestive gland, gill, foot, and gonad was obtained from each *M. galloprovincialis* individual from the injection and water tank cohabitation assay. In the cohabitation experiment, the number of individuals analyzed were 30 at T0, 10 from D, C^-^ and C^+^ samples and 30 from R samples at 1 and 4 days, for a total number of n = 150). In the bath experiment 30 individuals from T0 and 30 animals from both control and challenged group were analyzed at 1, 2, 3 and 4 days, for a total number of n = 270). After fixation in Davidson’s solution for 48–72 h (41), samples were dehydrated through a graded ethanol series, cleared with xylene (MYR STP 120, Especialidades Médicas Myr, Spain), and embedded in paraffin (MYR EC-350, Especialidades Médicas Myr). Serial sections (5 µm thick) were cut using a Leica RM2155 microtome (Leica Microsystems, Germany) and stained with hematoxylin and eosin (H&E) using automated staining equipment (MYR MYREVA SS-30, Especialidades Médicas Myr). For general evaluation and tissue description, the slides were examined under a light microscope (Leica DM LB, Leica Microsystems) and images were captured using an Olympus DP70 digital camera (Olympus Europa, Germany) and processed with Image Analysis software (Olympus Soft Imaging Solutions, Germany). Quantification was performed for the adipogranular cells in the soft connective tissue among digestive tubules, the goblet cells in the gill filaments and the free intervalvar hemocytes between gill filaments (n = 10 field/mussel) at each sampling time (0, 1 and 4 days post injection). Cell counts were performed within a standardized rectangular area measuring 100 × 1000 µm (0.1 mm²).

### 2.9 Data analysis

Data from experiments on hemocyte and hemolymph functional parameters are the mean ± SD of 3 hemolymph pools for C^-^, C^+^ and D (n = 3) and 6 hemolymph pools for R samples (n = 6) for the cohabitation experiment, and of 6 pools for each condition (n = 6) for the bath infection. Statistical analyses were performed by the non-parametric Kruskal-Walli’s test followed by Dunn’s test (*P* ≤ 0.05). Statistical calculations were performed using the GraphPad Prism version 7.03 for Windows, GraphPad Software, San Diego, CA, USA.

Quantification of different cell types in digestive gland and gills samples from the cohabitation experiment, was performed analyzing 10 optical fields per animal (see 2.8 for the number of mussels analyzed per condition). The measured variables were compared across groups and sampling time using R (version 4.3.0 in RStudio). As data did not meet normality assumptions, non-parametric tests were applied. A Kruskal-Wallis test was used to detect overall differences among experimental groups, followed by pairwise Dunn’s tests with Bonferroni correction. Superscript letters were assigned using the cld List function to indicate statistically significant differences (*P* < 0.05). Boxplots with group means and superscript letters were generated using the Minitab 18.1 software.

## 3 Results

### 3.1 Virulence of *V. ostreicida* at different temperatures

**Figure 2:**
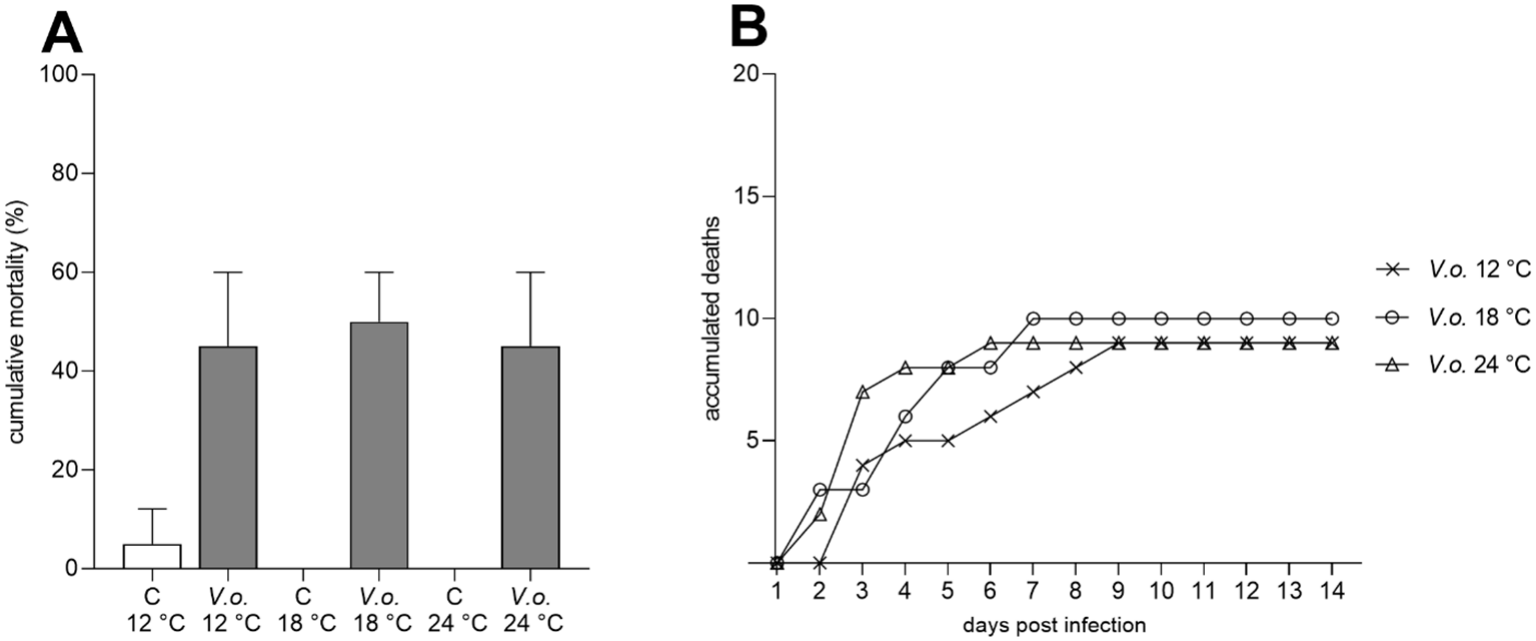
Effects of injection with *V. ostreicida* r172 (1 x 10^8^ CFU/100 µL; 100 µL/animal) on mussel mortality at different temperatures (12, 18 and 24°C). **(A)** Percent cumulative mortality at 14 days p.i. **(B)** Accumulated deaths over the duration of the experiment in mussels injected with r172; each line represents the sum of deaths from two independent assays performed at each temperature.

Infection with *V. ostreicida* r172 induced an average cumulative mortality of 45–50%, irrespectively of temperature, at 14 days p.i. (**Figure 2A**). Mortalities were recorded within 8 days p.i. in all groups, with no further increases up to 14 days (**Figure 2B**): however, the time course was affected by temperature, with maximal effects observed after 9, 7 and 6 days p.i., at 12, 18, and 24 °C, respectively (**Figure 2B**). In control mussels, mortality never exceeded 5%. PCR carried out on the cultures obtained from the whole tissue samples of dead mussels were all positive for *V. ostreicida*, confirming the recovery of the viable pathogen from them(not shown).

### 3.2 Detection of *V. ostreicida* in mussel samples from different aquaculture farms

The results of PCR analyses carried out in mussels sampled in April 2024 from different sites (**Supplementary Figure S1**) are reported in **Table 1**. The same data were obtained from both bacterial cultures of hemolymph pools (indicating the presence of viable bacteria) and individual mantles (where direct PCR analysis reveals the presence of both dead and live bacteria). All batch samples were positive for the presence of *V. ostreicida*; the highest value was detected in mussels from Fangar Bay, followed by those from Alfacs Bay. The lowest values were recorded in mussels from the open water farm in Alcanar; therefore, animals collected from this site were chosen for subsequent *in vivo* challenge experiments using two different experimental protocols of infection.

**Table 1:** Percentage (%) of *V. ostreicida* positive samples, evaluated by PCR, in mussels cultivated at different sites. Data represents the mean of both bacterial cultures of hemolymph pools and individual mantles.

Fangar Bay	Alfacs Bay	Alcanar
75%	40%	16%

### 3.3 Injection and cohabitation experiments

To investigate the effects of experimental infection of mussels with *V. ostreicida* and the possible horizontal transmission among individuals, a cohabitation experiment was first performed in May 2024 (see Methods for details). Mussels were injected with *V. ostreicida* (Donors-D, 10^8^ CFU/mL final nominal concentration) and placed in the same tanks with un-injected mussels (Recipients-R), in a 1D:3R ratio. Samples were collected at different times post-infection (day 0, 1, 4 and 20). Both negative (C^-^) and positive (C^+^) control samples were kept in separate tanks for the duration of the experiments.

#### 3.3.1 PCR detection of *V. ostreicida*

**Figure 3:**
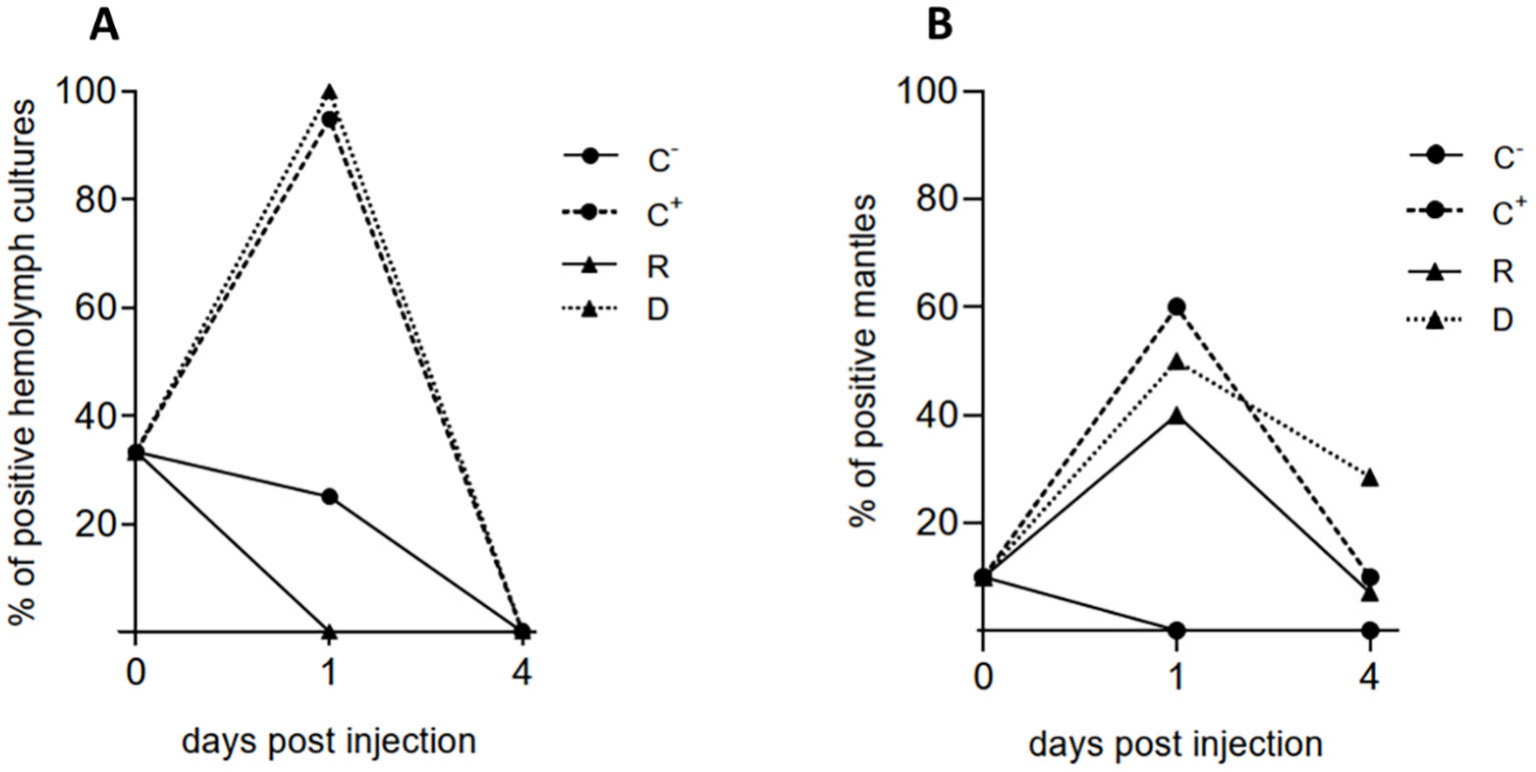
Detection of *V. ostreicida* by PCR in the cohabitation experiment in different experimental groups of mussels at different times post injection (day 0, 1 and 4). Negative (C-) and positive (C^+^) controls, donors (D), recipients (R). **(A)** Bacterial cultures from hemolymph pools; **(B)** individual mantle samples. Data are expressed as % positive samples in hemolymph pools (n=3 for C^-^, C^+^ and D samples; n=6 for R samples) and individual mantles (n=10 for C^-^, C^+^ and D samples; n=30 for R samples) (see Methods 2.5).

In bacterial cultures from hemolymph of mussels of the negative control group C^-^ (PBS-injected), *V. ostreicida* was detected in 33% of samples (**Figure 3A**). This value was higher than those recorded in the previous month (see **Table 1**). However, in these samples, a progressive clearance of live bacteria was observed over time. At day 1 p.i., both C^+^ and D samples were 100% positive to *V. ostreicida*, confirming the successful injection; however, no positive samples were detected in the R group. Total clearance of live *V. ostreicida* was observed at day 4 p.i. in all samples. PCRs were also run on bacterial cultures of individual water samples from different tanks. Only those containing injected animals (C^+^ and cohabitation tank) were positive to *V. ostreicida* at 1 day p.i.; at day 4 p.i., all water samples were negative (not shown).

PCR directly performed in individual mantle samples showed an initial detection of *V. ostreicida* in 10% of mussels at day 0. C^-^ samples showed a complete clearance from day 1 p.i. At day 1 p.i. *V. ostreicida* was detected in >50% of mantles from C^+^ and D samples. Moreover, 40% positive samples were detected in R group. An overall decrease in positive samples was observed at day 4 in all groups, indicating bacterial clearance also from this tissue, that was higher for C^+^ and R than in D samples (**Figure 3B**).

#### 3.3.2 Hemolymph functional parameters

**Figure 4:**
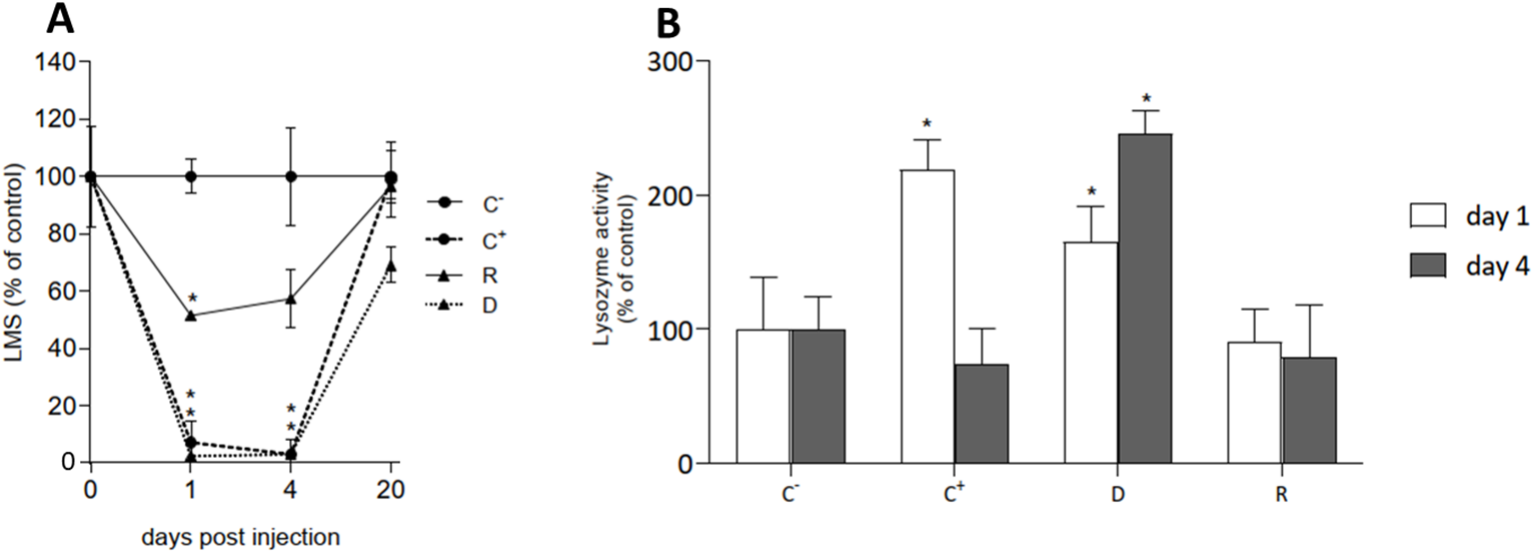
Hemolymph functional parameters in cohabitation experiment in different experimental groups of mussels at different times post injection (day 0, day 1 and day 4): negative (C-) and positive (C+) controls, donors (D), recipients (R). **(A)** Hemocyte lysosomal stability (LMS) and **(B)** serum lysozyme activity. Data are the mean ± SD evaluated in 3 pools for C^-^, C^+^ and D (n = 3) and 6 pools for R samples (n = 6). For more details see Methods 2.7. Statistical analyses were performed by non-parametric Kruskal-Wallis followed by Dunn’s multiple comparisons test, * = *P* < 0.05.

Hemocyte LMS and serum lysozyme activity were evaluated in hemolymph samples at different times p.i. and the results are reported in **Figure 4**. After 1 and 4 days a complete lysosomal destabilization (>95%) was observed in C^+^ and D samples, indicating irreversible cellular stress (**Figure 4A**). In these conditions, significant increases in lysozyme activity were observed (**Figure 4B**). A smaller but significant lysosomal stress was observed in R samples at 1 and 4 days p.i. (about -50% LMS with respect to the negative control), with no changes in serum lysozyme activity (**Figures 4A, B**). However, after 20 days, a full recovery of LMS values was observed and lysozyme activities were comparable with those observed at 1 and 4 days in all groups (data not shown).

#### 3.3.3 Histopathological analysis

**Figure 5:**
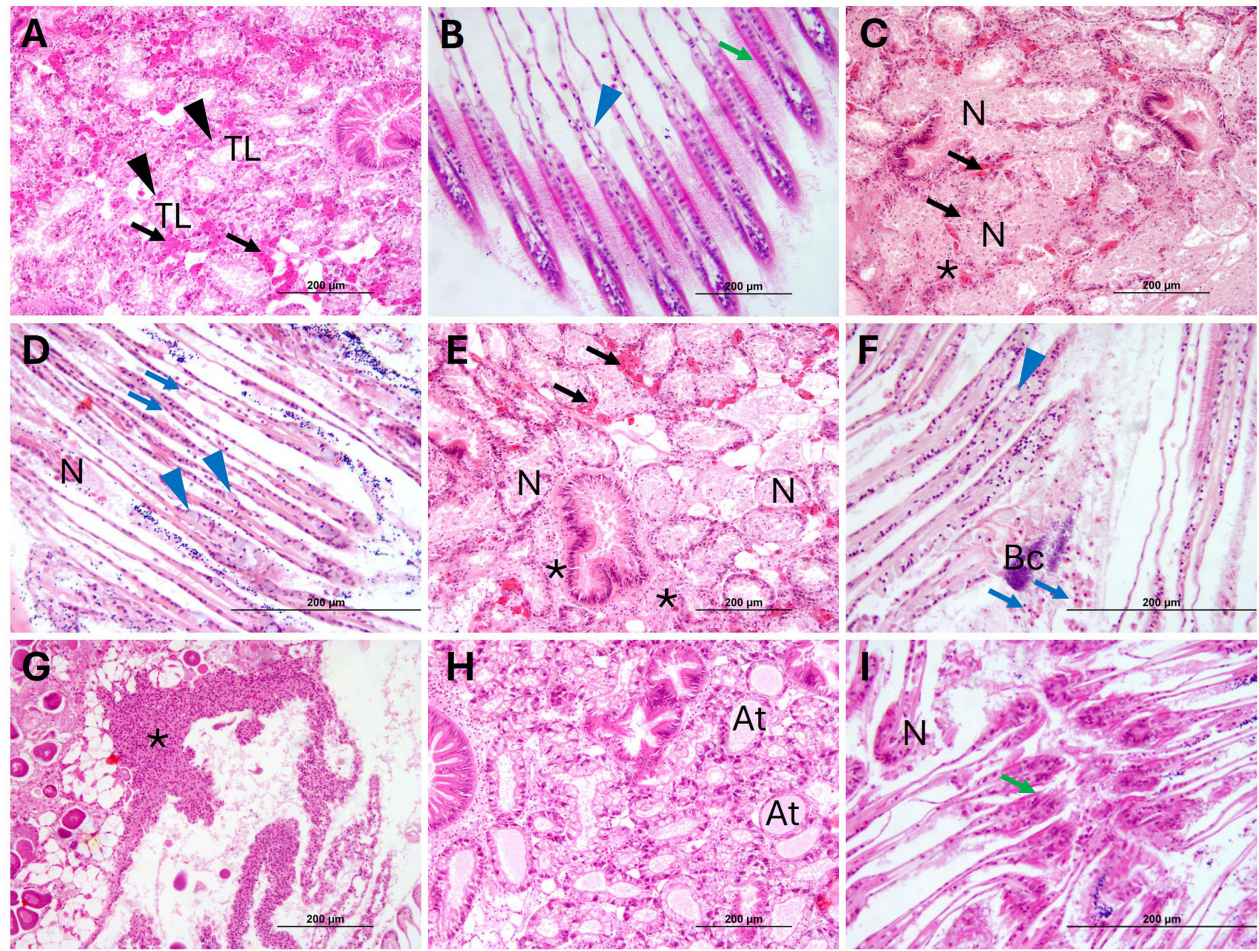
Representative H&E stain images of histological sections of *M. galloprovincialis* from the cohabitation experiment in negative controls, positive controls, donors and recipients at 1 day of exposure. **(A)** Digestive gland of negative control (C^-^) showing the tubular epithelium (black arrowheads) and ADG (black arrows). **(B)** Gill of C^-^. **(C)** Digestive gland of positive control (C^+^). **(D)** Gill of C^+^. **(E)** Digestive gland of Donor (D). **(F)** Gill of (D) **(G)** Hemolytic infiltrate (*) in soft connective tissue of (D) **(H)** Digestive gland of Recipient (R). **(I)** Gill of R. TL, Tubular lumen; N, Necrosis; At, Atrophied tubule; Bc, Bacterial colony; Blue arrowhead = goblet cell; Blue arrow = intervalvar hemocyte; Green arrow = Lamellar cilia. H&E Stain.

**Figure 6:**
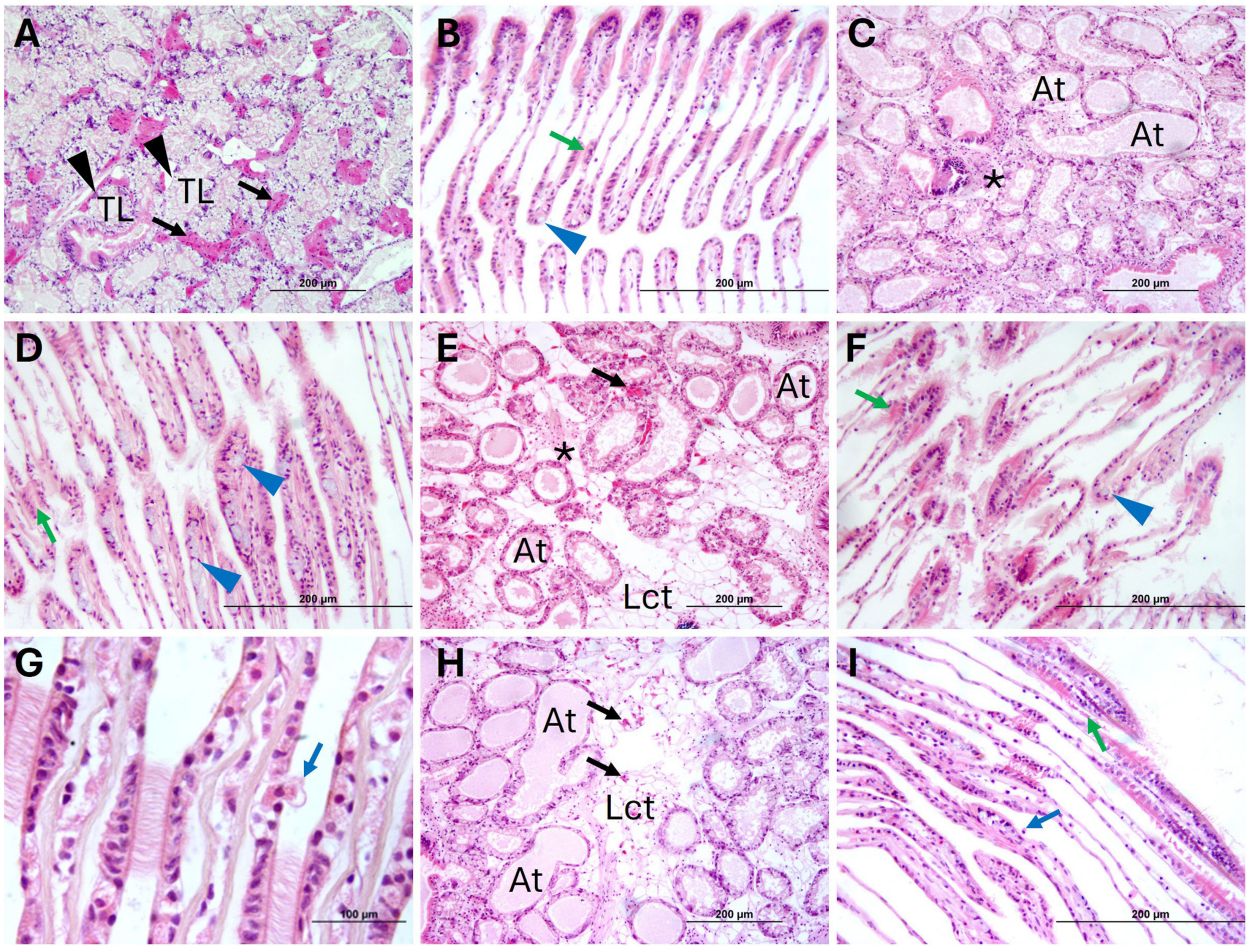
Representative H&E stain images of histological sections of *M. galloprovincialis* from the cohabitation experiment in negative controls, positive controls, donors and recipients at 4 days of exposure. **(A)** Digestive gland of negative control (C^-^) showing the tubular epithelium (black arrowheads) and ADG (black arrows). **(B)** Gill of C^-^. **(C)** Digestive gland of positive control (C^+^). **(D)** Gill of C^+^. **(E)** Digestive gland of Donor (D). **(F)** Gill of (D) **(G)** Hemocyte passing through the epithelium into the intervalvar space in (D) **(H)** Digestive gland of Recipient (R). **(I)** Gill of R. TL, Tubular lumen; At, Atrophied tubule. Lct, Loose connective tissue; * = hemocyte infiltrate; Blue arrowhead = goblet cell; Blue arrow = intervalvar hemocyte; Green arrow = Lamellar cilia. H&E Stain.

Representative images of tissue sections from mussels at 1 and 4 days p.i. are shown in **Figures 5** and 6, respectively. In negative controls (C^-^) no tissue alterations such as atrophy, hemolytic infiltration, or necrosis were observed at any sampling time in digestive gland and gills (**Figures 5 A, B**, **6A, B**). In the digestive gland, the tubules were closed together and displayed an irregular epithelium projecting into the lumen, with abundant vacuolation, indicating the presence of active, healthy digestive cells, and numerous adipogranular (ADG) cells in the intertubular spaces (**Figures 5A**, **6A**). The connective tissue showed a regular appearance and contained abundant ADG cells in most individuals. Also, the gills exhibited a normal appearance, with few free intervalvar hemocytes and intact interlamellar cilia (**Figures 5B**, **6B**).

At 1 day of exposure, C^+^ (70%), D (80%), and R samples (30%) exhibited varying degrees of lesions in both the digestive gland and gills. Multifocal necrosis, degeneration with epithelial sloughing into the tubular lumen, hemolytic infiltration and atrophy were observed in the digestive gland of C^+^ (**Figure 5C**), D (**Figure 5E**) and R (**Figure 5H**). Filament necrosis, epithelial sloughing, and interlamellar cilia damage were noted in the gills of C^+^ (**Figure 5D**), D (**Figure 5F**) and R (**Figure 5I**). In some mussels from C^+^ and D groups (**Figure 5G**), abundant hemolytic infiltration was observed in the loose connective tissue and mantle. In C^+^, 20% showed mild lesions, 40% moderate, and 10% severe. Among D, 20% exhibited mild lesions, 50% moderate lesions, and 10% severe lesions. In the R group, 23.33% of mussels exhibited mild lesions, and 6.67% showed moderate lesions; no individuals in this group showed severe lesions.

At 4 days p.i., mussels from all groups showed fewer lesions compared to those observed at day 1 (**Figure 6**). However, individuals from C^+^, D, and R groups exhibited some degree of digestive gland atrophy (50, 50 and 13.33%, respectively), with a reduction in the number of ADG cells and presence of hemocyte infiltration (**Figures 6C, E, H**), although no tissue necrosis was observed. In the gills, the presence of hemocytes into the inter-valvar space (**Figures 6G, I**), an increase in the number of goblet cells (**Figures 6B, D, F**) and damaged interlamellar cilia were observed; however, the tissue generally showed signs of recovery. In C^+^ and D 20% of the individuals exhibited mild lesions, while the remaining 30% showed moderate lesions. Among R, 10% of mussels exhibited mild lesions, and only 3.33% showed moderate lesions. No individuals showed severe lesions at 4 days of exposure.

**Figure 7:**
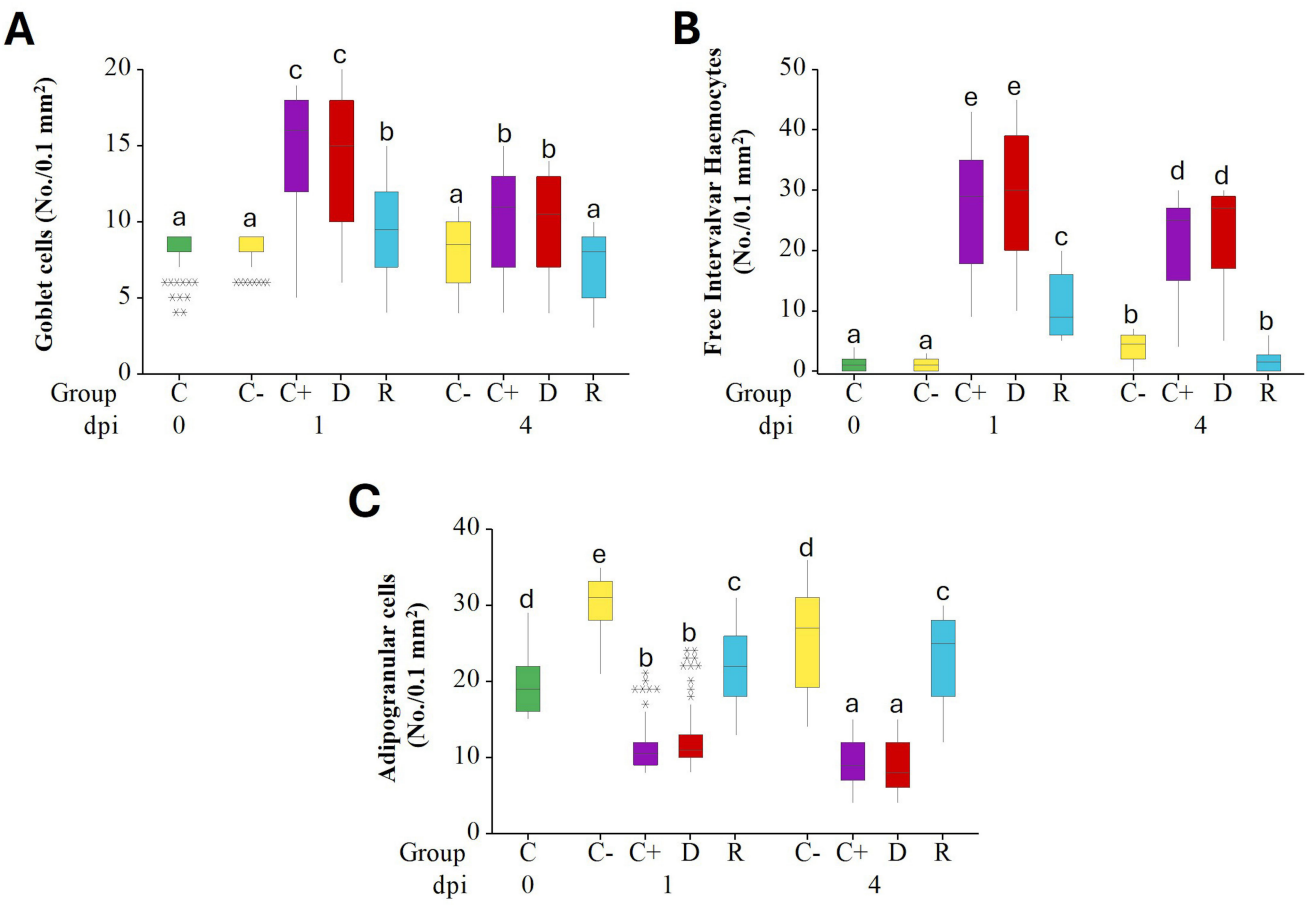
Boxplot graphs showing the number of goblet cells **(A)** and the free intervalvar hemocytes **(B)** in gill, and ADG(C) in digestive gland of *M. galloprovincialis* from the cohabitation experiment at 0, 1 and 4 days p.i. C_0_ = control at time 0; negative (C^-^) and positive (C^+^) controls, donors **(D)**, recipients (R). For each group, the number of individuals analyzed were: 30 mussels at T0, 10 from D, C^-^ and C^+^ samples and 30 from R samples at 1 and 4 days. Data were analyzed by the Kruskal-Wallis’ test, followed by pairwise Dunn’s tests with Bonferroni correction. Different superscript letters indicate statistically significant differences among groups. The line within each box represents the mean value; * = outlier points.

Quantitative analysis showed that at 1 day of exposure, C^+^, D and R samples exhibited a significant increase in the number of goblet cells in gills (**Figure 7A**) and free hemocytes in the inter-valvar space (**Figure 7B**), compared to the C^-^ group, while the number of ADG cells in the digestive gland decreased significantly (**Figure 7C**). However, at 4 days of exposure, the number of goblet cells and free hemocytes in the inter-valvar space in individuals from C^+^, D and R decreased significantly compared those observed at 1 day, approaching baseline values similar to those of the negative control C^-^ (**Figure 7**). In contrast, the number of ADG cells continued to decrease, at least in C^+^ and D groups, which showed the greatest degree of tissue damage.

### 3.4 Bath infection experiments

The effects of bath infection with *V. ostreicida* were evaluated in mussels sampled from the Alcanar site in July 2024. Samplings were performed from 1 to 4 days and the same parameters were evaluated as in injection/cohabitation experiments. No mortality was observed in any experimental group.

**Figure 8:**
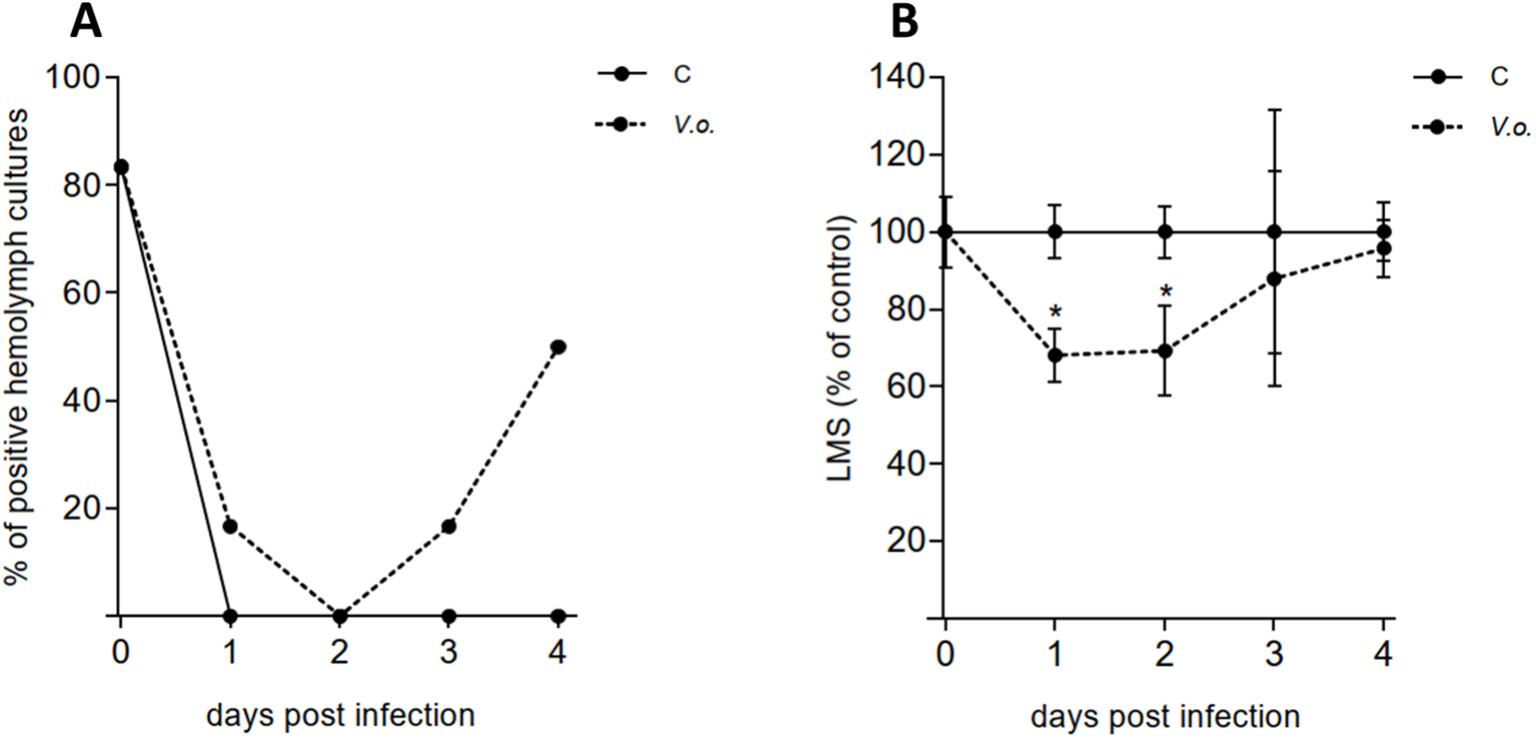
Bath infection experiment. Mussels were exposed to *V. ostreicida* spiked in water (10^5^ CFU/mL) and samples of hemolymph were analyzed from day 0 to day 4 post infection. C: control mussels and *V.o.: V. ostreicida* challenged mussels. **(A)** Detection of viable, cultivable *V. ostreicida* in hemolymph pools. PCR data are expressed as % positive samples in hemolymph pools (n = 6). **(B)** Hemocyte lysosomal membrane stability (LMS): data are the mean ± SD evaluated in 6 pools for each condition (n = 6). For more details about number of animals per pool see method 2.6. Statistical analyses were performed by non-parametric Kruskal-Wallis followed by Dunn’s multiple comparisons test, * = *P* < 0.05.

As shown in **Figure 8A**, in July the presence of *V. ostreicida* in hemolymph cultures at day 0, before infection, was detected in 5 out of 6 sample pools (80%), a value much higher than those recorded in April and May (see **Figure 3A**). In contrast, no *V. ostreicida* was detected in any mantle samples at any sampling time (data not shown). In control samples a complete clearance of cultivable bacteria from hemolymph was observed after 1 day. Bath infected mussels (*V.o.*) showed a slower elimination of *V. ostreicida* (reaching 100% clearance at day 2). However, a subsequent progressive increase in positive samples was observed in the following days (up to 50% at day 4), indicating successful infection. Water samples were positive for *V. ostreicida* growth at 2 days in Control tanks, and from day 2 to day 4 in *V.o.* infected tanks (data not shown).

No changes in hemocyte LMS were recorded in control samples throughout the experiment. In contrast, a small but significant decrease (>30%), indicating mild stress conditions, was observed in infected mussels from day 1, followed by full recovery by day 4 (**Figure 8B**). No changes in lysozyme activity were recorded (**Supplementary Figure S4**).

No histological alterations were observed in mussels analyzed at the different time points. There were no differences in the overall tissue appearance between the control group and the mussels exposed to *V. ostreicida*: both showed abundant ADG cells between the digestive tubules and within the loose connective tissue, star-shaped digestive tubules with numerous vacuolated epithelial cells, and gills without lesions. No hemolytic infiltration or necrosis was detected in any specimens. Representative images are reported at day 0, 1, 2 and 3 (**Supplementary Figures S2**, **S3**). Similarly, no histopathological alterations were observed at day 1 and 4 (not shown).

## 4 Discussion

In this work, first data are reported on the effects of *in vivo* experimental infections of adult *M. galloprovincialis* with the potential bivalve pathogen *V. ostreicida*, utilizing a strain (r172) previously isolated from a mussel mortality episode that occurred at Alfacs Bay in the Ebro delta (Spain) in 2022 (34).

The results of a virulence assay carried out in animals from another mussel site at the Ebro delta (Fangar Bay) indicate an average 50% mortality within 5 days of infection by intravalvar injection. Moreover, the present data show that *V. ostreicida*-induced cumulative mortality in experimental infection was independent of increasing temperatures. Actually, the mortality episode from which this strain was isolated occurred in April 2022 at 18 °C, a temperature that is not generally associated with mussel mortality outbreaks. However, the slightly faster onset of mortality observed at increasing temperatures suggests that seawater warming may contribute to a faster growing rate of this strain.

Mussels collected from different sites in the Ebro delta were then screened for the presence of *V. ostreicida*, utilizing the PCR protocol recently developed (34) with slight modifications. In April 2024 the scaling of the percentage of individuals positive for *V. ostreicida* was Fangar Bay > Alfacs Bay > Alcanar. Therefore, mussels collected from Alcanar, a site nearby open waters (Cases de Alcanar, Tarragona, Spain) were utilized for subsequent *in vivo* experiments utilizing two different models of infection, injection and bath exposure, respectively.

A month later (May 2024), a first injection/cohabitation experiment was carried out in order to investigate the time course of infection, the possibility of horizontal transfer of bacteria among individuals and mussel hemolymph parameters (see experimental set up in **Figure 1**). The results indicate that at 1 day p.i. bacterial cultures from hemolymph of injected mussel groups (C^+^ and D) were 100% positive for *V. ostreicida*, confirming successful infection. In contrast, no positive samples were detected in the R group, indicating the absence of transmission of live bacteria in hemolymph. By day 4 p.i. cultivable *V. ostreicida* was not detected in any sample group, indicating clearance of live bacteria from the circulation. Cultivable *V. ostreicida* was also detected in the water from both C^+^ and cohabitation tanks at 1 day p.i. but not at 4 days p.i. These data may reflect the results of an *in vivo* bactericidal activity of mussels against *V. ostreicida* over time. In contrast, in mantle samples, where direct PCR analysis reveals the presence of both live and dead vibrios, *V. ostreicida* was detected at 1 day p.i. in both D and R mussel groups (with 50 and 40% positive samples, respectively), indicating that bacterial transfer occurs among individuals of different groups, probably due to the direct contact of this tissue with the surrounding water. At 4 days p.i., the percentage of mantle positive samples declined in all groups, indicating partial elimination also from this tissue.

Infection with *V. ostreicida* induced parallel transient responses in hemolymph components. A dramatic decrease in hemocyte LMS was observed at 1 and 4 days in injected mussels (C^+^ and D) and a similar, although smaller effect, also in R samples. A parallel increase in serum lysozyme activity observed in C^+^ and D at 1 and 4 days p.i. may indicate enzyme leakage from damaged hemocytes. At 20 days p.i., full recovery of both parameters was observed in all samples. Due to the large decrease in hemocyte LMS observed in injected mussels at shorter times of exposure, indicating irreversible cell damage and death (36), the subsequent recovery of LMS values can be ascribed either to the presence in hemolymph of healthy hemocytes derived from differentiation of new granular hemocytes in the circulation, or recruitment of pre-existing hemocytes from tissues during recovery from stress conditions, or both (42).

The effects of infection with *V. ostreicida* and subsequent recovery, as well as the partial horizontal transfer from infected to uninfected mussels, were confirmed by histopathology data obtained in digestive gland and gills, showing varying degrees of lesions in different experimental groups and times of exposure. At 1 day p.i. multifocal necrosis, degeneration with epithelial sloughing into the tubular lumen, hemolytic infiltration and atrophy were observed in the digestive gland, along with filament necrosis, epithelial sloughing, and interlamellar cilia damage in the gills. These effects were recorded in the tissues of mussels from all experimental groups and were more severe in C^+^ and D with respect to the R group. Severe lesions (10%) were observed only in injected mussels, but not in the R group. At 4 days p.i. both tissues showed signs of recovery, and no severe lesions were observed in any experimental group. In more detail, quantitative differences could be observed in the number of gill goblet cells and intervalvar (pallial) hemocytes, showing a transient increase at 1 day p.i. in both injected and R groups, followed by partial recovery at 4 days. Goblet cells are responsible for mucus secretion, that represents a first defense reaction in epithelia (43).

**Figure 9:**
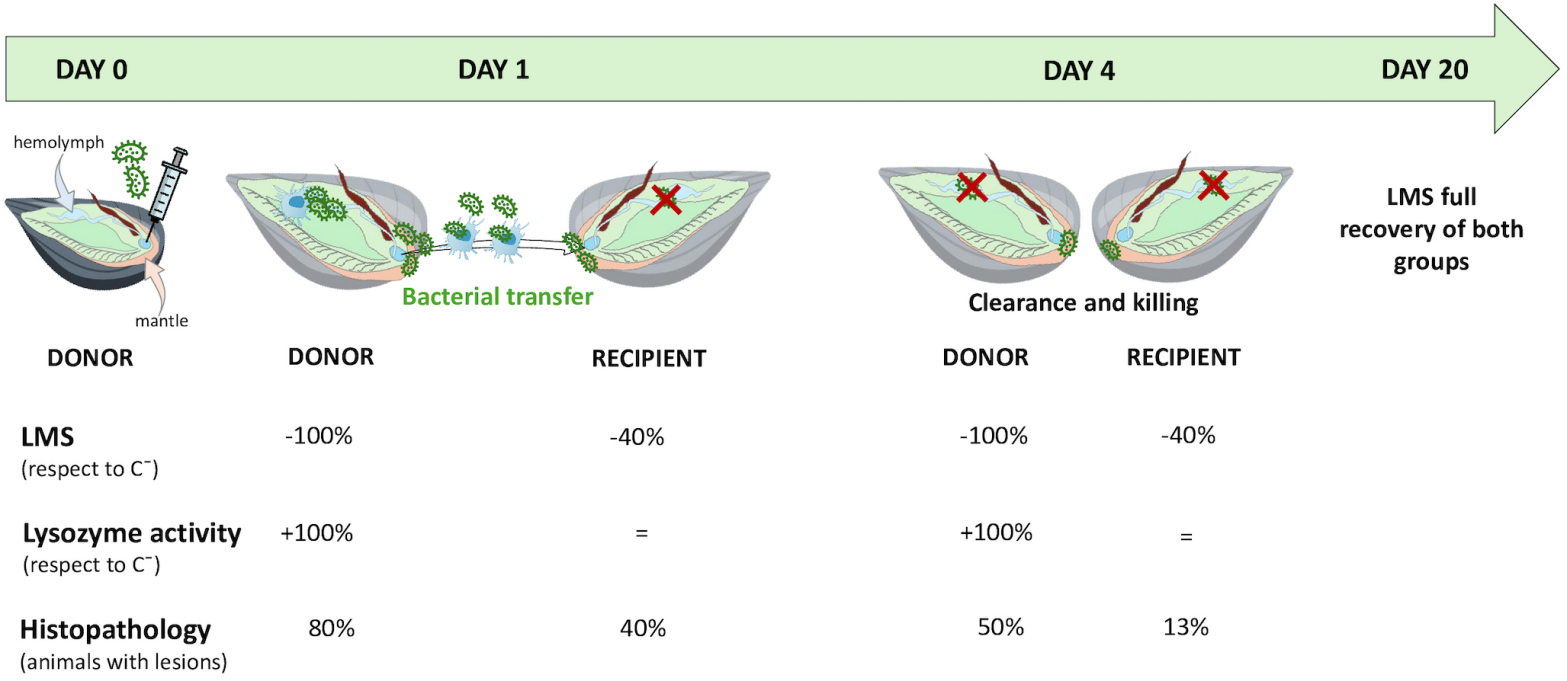
Summarized results from cohabitation experiment.

Bivalve hemocytes are able to move freely in a semi-open circulatory system, reaching the surface of the body and the extrapallial cavity: several studies underlined bidirectional exchanges between circulatory and pallial hemocytes (44–46). Since the pallial surfaces (epithelial tissues associated with the gills, mantle and palps) are directly exposed to the surrounding environment, and thus to the multitude of micro-organisms present in the water column, hemocytes associated with pallial organs represent the first host immune cells that can potentially interact with waterborne bacteria, and thus have major implications for the mechanisms of early host-pathogen interactions. In mussels, it has been shown that in response to temperature stress, hemocytes are released in seawater, where they can survive and remain functionally competent before entering other mussels; moreover, opportunistic bacteria can infect hemocytes in seawater and take advantage of the entry of hemocytes into other mussels to spread into healthy individuals (45). The transient increase observed in the number of intervalvar hemocytes in both injected and recipient mussels underlines a possible role for this hemocyte sub-population in response to mussel infection with *V. ostreicida*, and suggests a possible role in horizontal transfer of these vibrios among individuals (see **Figure 9**). Although mussel intervalvar hemocytes have been characterized for functional and molecular properties (46) further studies are needed on their role in spreading pathogen infection under different environmental stressors.

Finally, the results underline a persistent decrease in digestive gland ADG cells in injected mussel groups. ADG are storage cells present in different bivalve tissue containing lipid and glycogen reserves, that are essential for providing the energy and resources for maintaining bivalve health (47, 48). These data indicate the utilization of energy reserves to support defense responses during mussel infection with *V. ostreicida.*

Overall, the injection/cohabitation experiments indicate the possibility of horizontal transfer of *V. ostreicida* from infected to uninfected mussels. In this process, where intervalvar hemocytes may play a role (45, 46), the proximity of cultivated mussels at different sites may represents a significant risk of infection. Such a transfer has been previously described in cohabitation experiments of infected oysters and mussels, with stronger consequences on recipients mussels (31, 38). However, the results here obtained cannot be compared with those of previous studies, due to several differences in experimental set up and endpoints measured and, most of all, to the utilization of *V.* sp*lendidus* strains, that represent known bivalve pathogens (31, 38).

A bath infection experiment was then carried out in July 2024, exposing mussels to *V. ostreicida* at 10^5^ CFU/mL in seawater. At this time of the year, the mantle tissues of mussels from Alcanar were negative for the presence of *V. ostreicida*; although in hemolymph cultures at the beginning of the experiment a high percentage of positive samples was recorded (80%), this value declined to 0% from 1 day of exposure in clean seawater in control (unexposed mussels) and from day 2 in exposed mussels, indicating a complete clearance of cultivable bacteria from hemolymph of control and infected mussels. However, a subsequent rise in bacterial growth was observed in the hemolymph of infected mussels, as a result of the filtration process of water by the gills and subsequent transfer of live bacteria to the circulation. Actually, water samples were positive for cultivable bacteria from day 2 to day 4 in the *V. ostreicida* infected tank, despite the daily water change, suggesting release of live bacteria from animals. LMS data indicate a fast and transient stress response in circulating hemocytes from 1 day of exposure to *V. ostreicida*. In mussel hemocytes, where LMS is an extremely sensitive parameter of cellular stress, such an effect could be induced by both cultivable and not cultivable or dead bacteria.

Finally, bath infection did not result in an increase of *V. ostreicida* positive samples in the mantle, or significant histopathological alterations in either gills or digestive gland. Although further data on the presence of live, cultivable *V. ostreicida* r172 in the sea water at the site of mussel collection will complement those on the positivity of mussel samples to this strain, the results indicate a fast depuration process from mussel tissues.

The results obtained from injection experiments demonstrate that *V. ostreicida* induces significant stress conditions in *M. galloprovincialis*, as shown by hemolymph parameters and tissue histopathology. However, all the observed responses were transient, and paralleled by a decrease in the presence of *V. ostreicida* within 4 days, indicating a capacity of the mussels to partially cope with infection. Such a capacity was confirmed in the more environmentally realistic exposure conditions of bath exposure experiments, that resulted in lower infection and stress conditions. The results of both *in vivo* experiments show that LMS represents a sensitive parameter to measure the response of mussel hemocytes to *V. ostreicida*, independent of the route of infection.

Overall, the results indicate a moderate pathogenicity of *V. ostreicida* r172 to mussels in *in vivo* infection models. These data provide further information on the interactions between the bivalve host and potential pathogenic vibrios. For *Mytilus* spp., these data may help understanding or predicting the onset and progression of disease in the context of increasing mortality outbreaks of farmed mussels in different environmental contexts.

From the present study, two additional points emerge that warrant further consideration.

The first concerns the background infection with cultivable *V. ostreicida* observed in hemolymph samples of control mussels used for laboratory experiments. Although mussels were initially collected from the site with the lowest *V. ostreicida* contamination (Alcanar), a progressive increase in the percentage of positive samples was recorded (from 16% in April to 33% in May and 80% in July) corresponding to seawater temperatures at the time of collection of 15.9, 18.7, and 24.9°C, respectively. While this variation could reflect natural differences among mussel groups, the data suggests that the presence of cultivable *V. ostreicida* in hemolymph increases with rising seawater temperature. In this context, the application of PCR to bacterial cultures from tissue samples may greatly enhance monitoring of *V. ostreicida* dynamics in mussel tissues under field conditions throughout the year.

With regards to the effects of *V. ostreicida* on mussel mortalities, significant effects were recorded in virulence assays carried out in mussels sampled from Fangar Bay in winter; however, no mortality was observed in subsequent injection and bath infections experiments carried out on mussels from Alcanar, a site from nearby open waters, sampled in late spring. The results indicate a distinct susceptibility of different populations of cultivated mussels, irrespectively of seawater temperatures recorded at sampling times. This may depend on other several factors, including salinity fluctuations, tissue loads of contaminants, mussel density and depth in the long line system (5), as well as to previous exposure history of mussels to other vibrios, leading to immune priming (42, 49). From the present data, temperature does not seem to represent a major conditioning factor in influencing the virulence of *V. ostreicida*; however, as underlined above, environmental temperatures may influence the presence of live bacteria in mussel hemolymph. Finally, since mussel stocks in the Ebro delta cultivation areas are imported from other growing sites, their genetic background would also play a role in resistance to infection (50).

Overall, the results underline the complexity of understanding the infection dynamics of environmental vibrio strains and their outcome in mussel populations.

## Data Availability

The raw data supporting the conclusions of this article will be made available by the authors, without undue reservation.
