# Supplementary material for: Dynamics of Vibrio ostreicida in Mytilus galloprovincialis through in vivo infections
Source: Front Immunol. 2025 Nov 26;16:1727164. doi: 10.3389/fimmu.2025.1727164 (PMC12689316; doi:10.3389/fimmu.2025.1727164)
Supplement: Supplementary file 1 [file Table1.docx]

Supplementary Material to

Dynamics of *Vibrio ostreicida* in *Mytilus galloprovincialis* through *in vivo* infections

Martina Leonessi^1,2*^, Jose R. Lopez^3,4^, Yanis Cruz-Quintana^3,5^, Manon Auguste^1,2^, Karl B. Andree^3^, Margarita Fernandez-Tejedor^3^, Luigi Vezzulli^1,2^, Dolors Furones^3^, Laura Canesi^1,2^

^1^Department of Earth, Environmental and Life Sciences (DISTAV), University of Genoa, Genoa 16132, Italy

^2^National Biodiversity Future Center, Palermo, Italy

^3^IRTA, Aquaculture Program. La Ràpita, Spain

^4^Universidade de Santiago de Compostela, Santiago de Compostela, Spain

# ^5^Facultad de Acuicultura y Ciencias del Mar, Universidad Técnica de Manabí, Bahía de Caráquez 130104, EcuadorMaterial and methods

**Evaluation of hemolymph functional parameters**

All procedures were performed as previously described (1). Functional parameters were evaluated directly on hemolymph samples after challenge. Hemolymph was extracted from the posterior adductor muscle using a sterile 1 mL syringe with an 18 G1/2” needle, filtered through sterile gauze and pooled in Falcon tube at 18 °C as previously described. Hemocyte monolayers were prepared depositing 20 µL drops of whole hemolymph on microscope slides and the cells were let to adhere at 18 °C for 20 min before removing the excess hemolymph excess (1). LMS was evaluated in hemocyte monolayers by the Neutral Red Retention Time (NRRT) assay (2) as previously described (1,3). After the adhering time, samples were incubated with a neutral red (NR) (Sigma-Aldrich, Milan, Italy) solution (40 µg/mL in ASW). After 15 min, excess dye removed, a drop of ASW was added and the slides were observed under an optical microscope from time zero every 15 min. The percentage of cells showing loss of dye from lysosomes in each field (10 fields each containing 8-10 cells) was evaluated until 50% of the cells showed sign of lysosomal leaking. All incubations were carried out at 18 °C. For determination of lysozyme release aliquots of whole hemolymph were centrifuged for 10 min at 100 x g, 4 °C and the supernatants collected and frozen at -80 °C. Lysozyme activity was evaluated in the extracellular medium spectrophotometrically at 450 nm using a suspension of *Micrococcus lysodeikticus* (15 mg/100 mL in 66 mM phosphate buffer, pH 6.4). Protein content was determined using the Bradford method using bovine serum albumin (BSA) as a standard. Data are reported per mg/protein sample and expressed a percentage of control values.

**PCR protocol**

PCR amplifications were carried out in a mixture containing 0.5 U Q5 High-Fidelity DNA Polymerase (Biolabs), 1X Q5 Reaction Buffer (containing 2 mM Mg^+2^), 200 µM of dNTPs, 0.5 µM of each primer, 1X of Q5 High GC Enhancer and Ultra Pure PCR water as previously described (4) using primers shown in **Table S1**. Optimal conditions for PCR reaction were chosen as follows (**Table S2**): an initial denaturation at 98 °C for 30 s followed by 35 amplification cycles of denaturation at 98 °C for 10 s, annealing at 55 °C for 30 s, extension at 72 °C for 30 s and a final elongation at 72 °C for 2 min. DNA from type strain *V. ostreicida* CECT7398T at 5 ng/µL was used as positive control, and Ultra Pure PCR water was employed as a negative control. Samples of PCR products were run on a 1.5 % agarose gel TAE (Tris-acetate-EDTA buffer) stained with ethidium bromide. The Invitrogen 1Kb Plus Ladder was used as a molecular weight marker.

# Tables

**Table S1**. Primer sequences for amplification of *V. ostreicida* and the size of the obtained products.

| **Gene** | **Primers sequences from 5' to 3'** | **product size** | **Reference** |
| --- | --- | --- | --- |
| *gyr B* | F: GACAACGGGAACAAAAATCC | 491 bp | Lopez et al., 2025 |
|  | R: ACACGACCGCGGTTAGGC |  |  |

**Table S2**. PCR conditions

| **DNA concentration** | mantles: 50 ng/µL  bacterial cultures: 5 ng/µL |
| --- | --- |
| **N° of cycles** | 35 |
| **Denaturation 1** | 98 °C x 30 s |
| **Denaturation 2** | 98 °C x 10 s |
| **Annealing** | 55 °C x 30 s |
| **Extention** | 72 °C x 30 s |
| **Final elongation** | 72 °C x 2 min |

# Figures


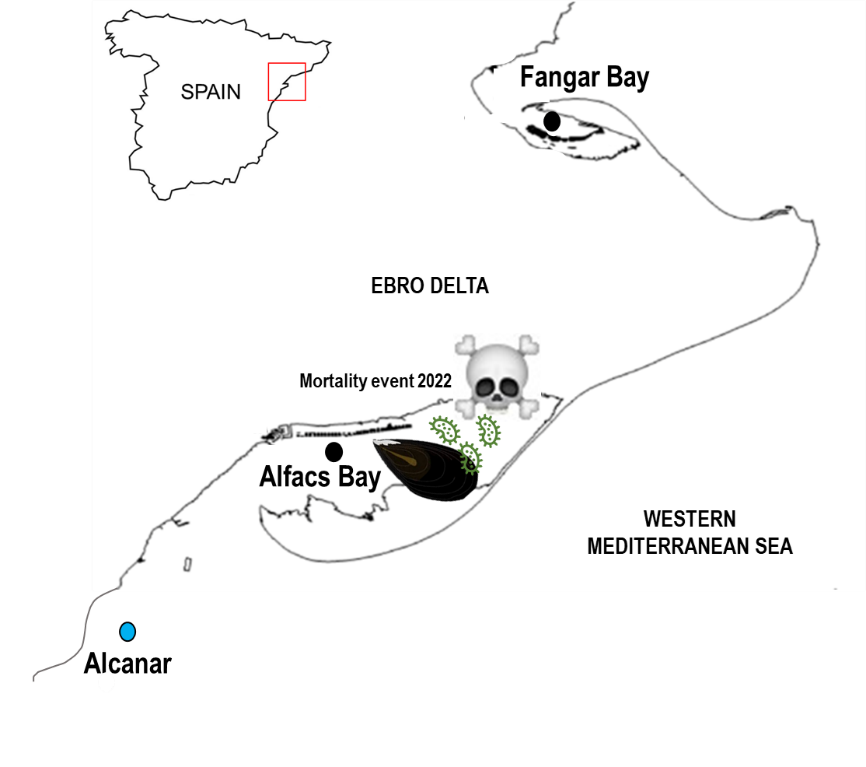


**Figure S1**. Locations of the 3 mussel farms in Ebro Delta (Spain): Fangar Bay, Alfacs Bay and Alcanar.

**
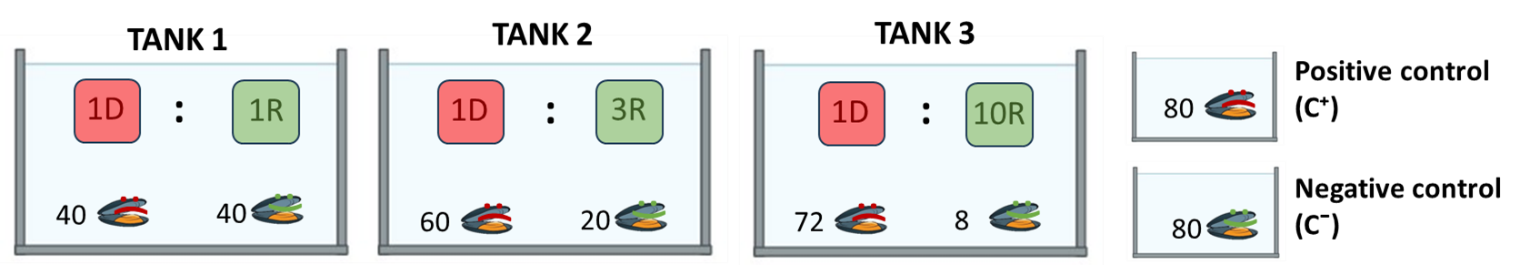
**

**Figure S2**. Experimental conditions in the preliminary cohabitation experiments. R: recipients, D: donors. 1D:1R (Tank 1), 1D:3R (Tank 2) and 1D:10R (Tank 3). Positive (*V. ostreicida* injected) (C^+^) and negative (PBS injected) (C^-^) controls were run in parallel. The number of D and R mussels in each tank is indicated, with a total of 80 mussels per each tank. The experiment was run twice. During the experiments, animals were maintained under static conditions in UV-treated and filtered seawater at 18 °C with aeration. The mortality in each tank was monitored daily, dead mussels were removed, and water was changed daily.

# Results

**
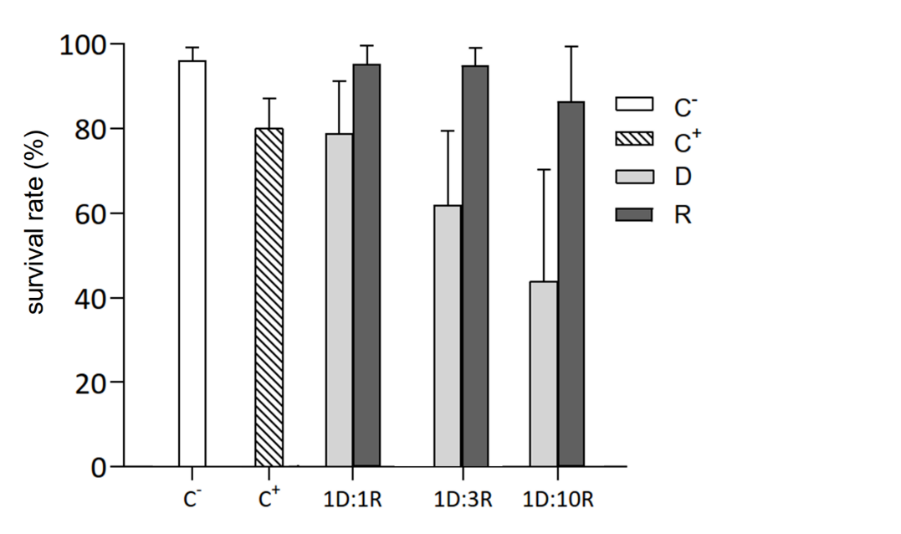
**

**Figure S3**. Survival rate (%) during the cohabitation experiments up to 20 days in Donors (D) and Recipients (R) in the different tanks: 1D:1R (Tank 1), 1D:3R (Tank 2) and 1D:10R (Tank 3). The experiment was run twice. C^-^: negative control, C^+^: positive control, R: recipients, D: donors.


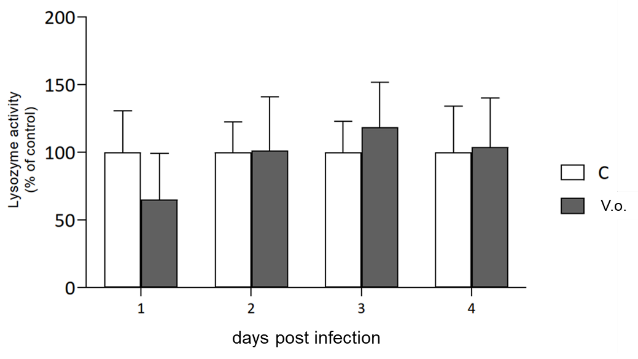


**Figure S4**. Effects of bath infection on lysozyme activity in hemolymph of *M. galloprovincialis*. Data, representing the mean ± SD of 6 pools of hemolymph samples for each condition (n=6), were analysed by non-parametric Kruskal-Wallis followed by Dunn’s multiple comparisons test. No significant differences were observed among different groups (p > 0.05).


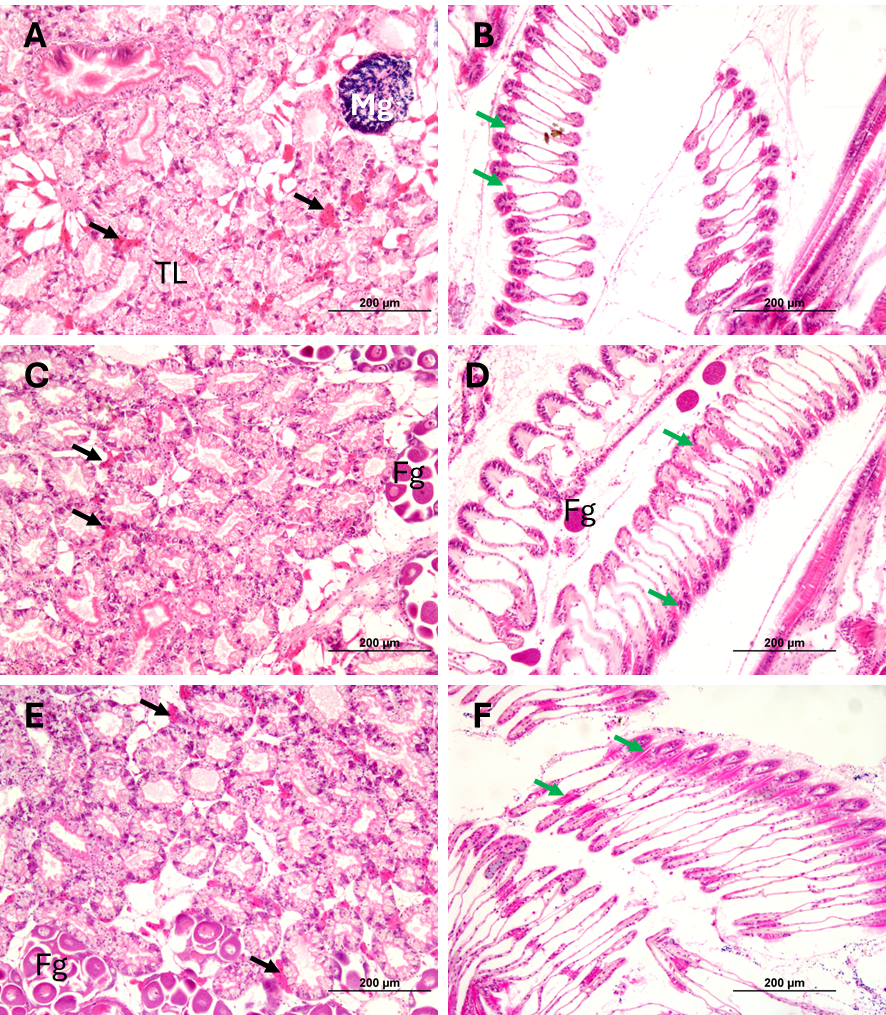


**Figure S5**. Representative histological sections of *M. galloprovincialis* from control and *V. ostreicida*-challenged groups in the bath infection experiment at 0 and 1 day of exposure. A) Digestive gland of *M. galloprovincialis* at time 0 (prior to immersion) showing the adipogranular cells (black arrows) among tubular lumen (TL) and adjacent male gametes (Mg). B) Gill of the same specimen. C) Digestive gland of a mussel from the control group after 1 day of exposure. D) Gill of the same specimen. E) Digestive gland of a mussel from the *V. ostreicida*-challenged group after 1 day of exposure. F) Gill of the same specimen. TL = tubular lumen; Fg = female gametes; Mg = male gametes; Black arrow = adipogranular cells; Green arrow = lamellar cilia. H&E Stain.


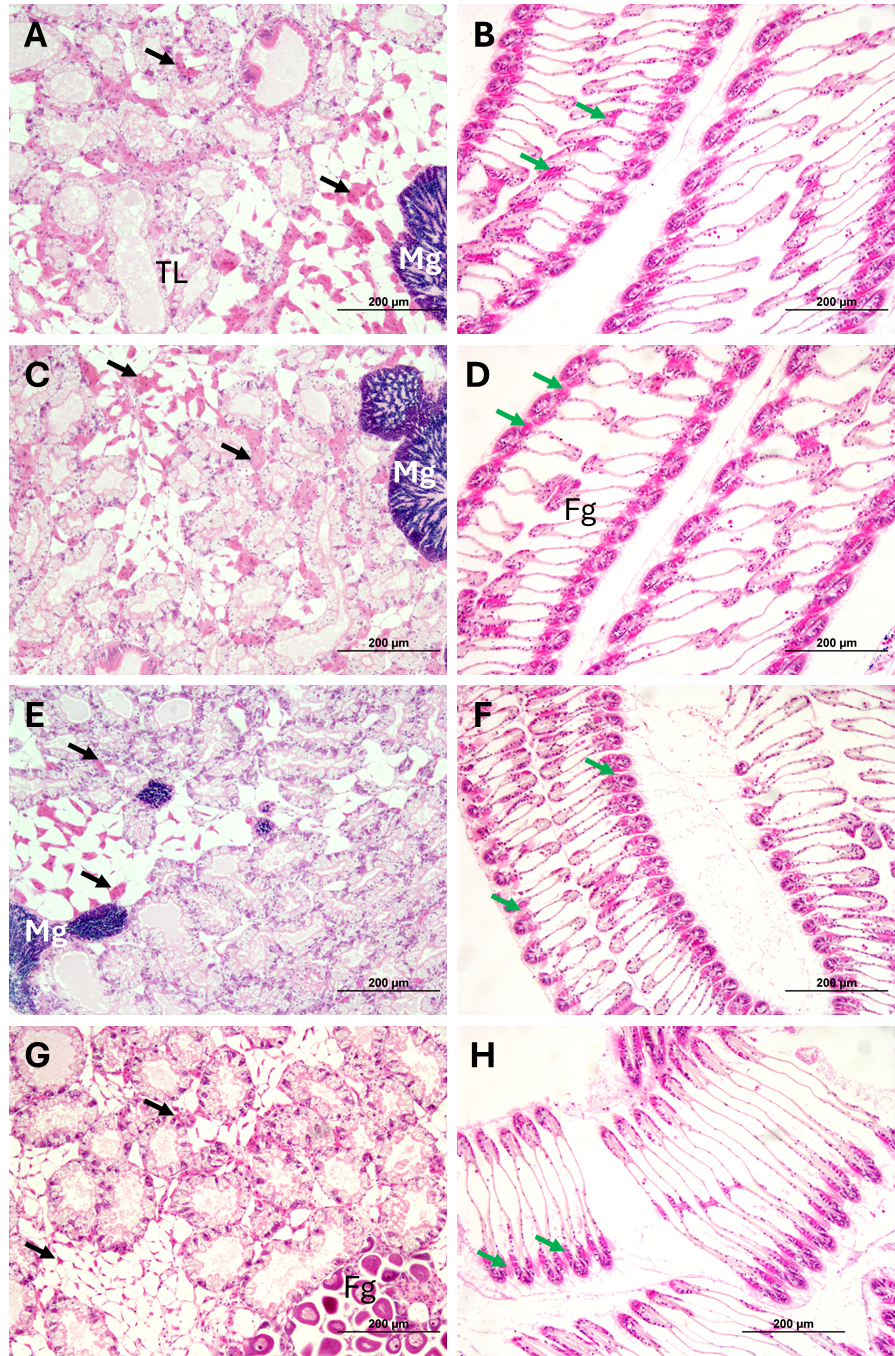


**Figure S6** - Representative histological sections of *M. galloprovincialis* from control and *V. ostreicida*-challenged groups in the bath infection experiment at 2 and 3 day exposure. A) Digestive gland of a mussel from the control group after 2 days of exposure. B) Gill of the same specimen. C) Digestive gland of a mussel from the *V. ostreicida*-challenged group after 2 days of exposure. D) Gill of the same specimen. E) Digestive gland of a mussel from the control group after 3 days of exposure. F) Gill of the same specimen. G) Digestive gland of a mussel from the *V. ostreicida*-challenged group after 3 days of exposure. H) Gill of the same specimen. TL = Tubular lumen; Fg = Female gametes; Mg = Male gametes; Black arrow = Adipogranular cells; Green arrow = Lamellar cilia. H&E Stain.

# Bibliography

1. Auguste M, Rahman FU, Balbi T, Leonessi M, Oliveri C, Bellese G, et al. Responses of Mytilus galloprovincialis to challenge with environmental isolates of the potential emerging pathogen Malaciobacter marinus. Fish Shellfish Immunol. 2022 Dec;131:1–9.

2. OSPAR Commission. Background documents and technical annexes for biological eﬀects monitoring. In 2013. p. 239. Available from: www.ospar.org.

3. Auguste M, Leonessi M, Bozzo M, Risso B, Cutroneo L, Prandi S, et al. Multiple responses of Mytilus galloprovincialis to plastic microfibers. Sci Total Environ. 2023 Sep;890:164318.

4. Lopez JR, Leonessi M, Fernandez-Tejedor M, Auguste M, Doni L, Cruz-Quintana Y, et al. First description of Vibrio ostreicida as putative causal agent of disease in Mediterranean mussel (Mytilus galloprovincialis) and development of a PCR protocol for its rapid diagnosis. J Invertebr Pathol. 2025 Sep;212:108399.
